# GENETIC ANALYSIS 21 SHORT TANDEM REPEATS (STR) LOCUS IN MINANGKABAU POPULATION, WEST SUMATERA, INDONESIA

**DOI:** 10.21010/Ajid.v16i2.4

**Published:** 2022-05-06

**Authors:** Citra Manela, Rika Susanti, Djong Hon Tjong, Ahmad Yudianto

**Affiliations:** 1 Department of Forensic Medicine, Faculty of Medicine, Andalas University, Padang , Indonesia; 2 Department of Biology, Faculty of Mathematics and Natural Sciences, Universitas Andalas, Padang, Indonesia; 3 Forensics and Medicolegal Department, Faculty of Medicine, Universitas Airlangga, Surabaya-Indonesia; 4 Human Genetic Study Group, Institute of Tropical Diseases, Universitas Airlangga, Surabaya, Indonesia; 5 Magister of Forensics, Postgraduate School, Universitas Airlangga, Surabaya, Indonesia

**Keywords:** allele frequency, Minangkabau, short tandem repeats

## Abstract

**Background:**

Minangkabau is the majority ethnic group in West Sumatra, Indonesia. West Sumatra is a disaster area, especially earthquakes and the potential for a tsunami. Allele frequency for 21 short tandem repeat locus and genetic variation are not well known. This data is essential for calculating the Paternity Index and Match Probability for forensic identification.

**Materials and methods:**

This was an observational study. We analyze the GlobalFiller STR loci in 25 unrelated individuals from Minangkabau ethnic group. The DNA was extracted using a Prefiller kit and amplified with a Global Filler kit by a GeneAmp PCR System, followed by capillary electrophoresis using ABI Prism 3500 Genetic Analyzer. Data analysis was performed by using Easy DNA and FORSTAT software.

**Results:**

We observed 162 alleles with allele frequencies between 0.02 – 0.36. The highest expected heterozygosity and the highest power of discrimination were at the SE33 loci, and the highest match probability was at the D2S441 locus. The Chi-square test showed that all STR loci followed Hardy–Weinberg equilibrium (p > 0.05). All loci were highly polymorphic (PIC > 0.5). The combined discrimination capacity of each locus in the population was 99,999%.

**Conclusion:**

The 21 STR loci are useful for forensic analysis and population genetic studies of the Minangkabau population.

## Introduction

Indonesia, a maritime nation comprising over 17 000 islands straddling the Pacific and Indian Oceans, links mainland Asia with the Pacific world. Relative to its land area, Indonesia is one of the most varied regions regarding ethnic, linguistic, and genetic diversity (Tumonggor *et al.*, 2013).

Minangkabau is the majority ethnic group in West Sumatra, Indonesia. West Sumatra has an area of 42,013 km, and which total population is 5,580,232 people. Earthquakes often strike along Sumatera’s west coast, both on land and beneath the sea. The high level of earthquakes, especially those that occur under the sea, has the potential to cause a tsunami. (BPS Sumbar, 2021).

**Figure 1:**
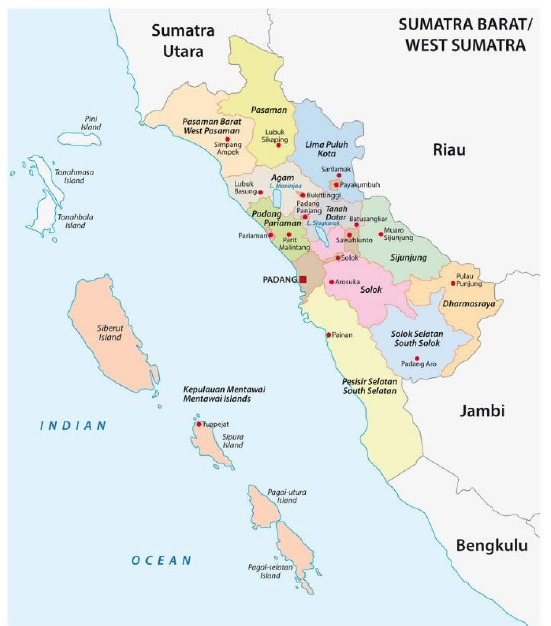
A map showing the location of the studied Minangkabau population in West Sumatera Province, Indonesia

Short tandem repeats (STR) are a repeated DNA sequence with a 2-7 bp core repeat unit. The number of repeats varies significantly between individuals. As a result, it is advantageous in forensic identification (Butler John M, 2010). The 13 CODIS (Combined DNA Index System ) core loci (D8S1179, D21S11, D5S818, CSF1PO, D3S1358, TH01, D13S317, D16S539, TPOX, D18S51, vWA, D7S820, FGA) have been officially included in DNA databases in the United States since 1998. They have recently enlarged to 20 with seven more STRs (D1S1656, D2S441, D2S1338, D10S1248, D12S391, D19S433, and D22S1045) (Karantzali *et al.*, 2019). The GlobalFiller STR kit was created by upgrading the 13 CODIS loci to 20 loci and was made available to the laboratories.

Since the last locus of CODIS is located within the ESS (European Standard Set of Loci), the GlobalFillerSTR kit has become a global system combining CODIS and ESS regions. The GlobalFiller STR kit was used in our study because of these distinguishing features.

The first stage in DNA analysis is to determine the DNA profile from the biological samples and the interpretation of data obtained from this DNA. To interpret the evidence obtained from the DNA molecule correctly, it is necessary to know how often genetic signs are seen in the relevant population (Canpolat *et al.*, 2021). The variety and frequency of alleles at each locus can only be determined through studies conducted on each population. So, the allele frequencies of genetic markers used in forensic sciences should be determined for each population, and a database should be established. It is critical to use an accurate society database in the statistical calculations made to evaluate the results of DNA analysis (Goodwin, W., Linacre, A., Hadi, 2007). The study of genetic analysis STR loci of the Minangkabau ethnic group is not widely known.

## Materials and methods

Whole blood samples were obtained from 25 unrelated healthy individuals of the Minangkabau population. Informed consent was obtained from all individuals. This research received ethical approval from the research ethics committee of the Faculty of Medicine, Universitas Andalas 414/UN.16.2/KEP-FK/2021.

Genomic DNA was extracted with PreFiller according to a protocol. The DNA quantification process was performed by dripping 1 μl of the DNA sample on the NanoVueTM Plus reading device (GE Healthcare, USA). Making a master mix in a 0.2 ml Eppendorf tube containing 0.5 μl AmpFISTR PCR reaction mix, 0.5 μl AmpFISTR Gold DNA Polymerase, and 5.5 μl AmpFISTR Globalfiller Primer Set is the first step in the amplification method. In a 0.2 ml Eppendorf tube, 15 μl of the master mix was added, followed by 1 μl of DNA and 9 μl of pH 8.0 TE buffer solution until the total volume was 25 μl. Furthermore, for each amplification process, positive and negative controls were used. The negative control contained 15 μl of the master mix and 10 μl of pH 8 buffer TE.

The positive control included 15 μl of the master mix and 10 μl of DNA template (Globalfiller kit). The amplification of each sample, positive and negative controls, was carried out using the GeneAmp PCR System 9700 (Applied Biosystem, USA). The stages of the amplification process carried out were initial heating at 95°C for 11 minutes, followed by 28 cycles, denaturation at 94°C for 1 minute, annealing at 59°C for 1 minute, elongation at 72°C for 1 minute, and final elongation at 60°C for 60 minutes. The amplification of each sample was repeated two times (Thermo Fisher Scientific, 2019).

A total of 1 μl of the amplified sample was placed in a 96-well plate (Applied Biosystems, USA) containing 9.6 μl of HiDi formamide and 0.4 μl of LIZ Size Standard. The well plate was centrifuged for 1 minute at 3000 rpm. Using a thermocycler, GeneAmp PCR System 9700 (Applied Biosystem, USA), samples were denatured at 95^0^ C for 2 minutes before being cooled in a freezer at 20^0^C for 3 minutes. Allele readings at the amplified loci were performed by the ABI PRISM 3500 Genetic Analyzer (Applied Biosystem, USA) and analyzed using Genemapper ID-X v1.4 software (Applied Biosystem, USA) (Applied Biosystems, 2010).

Easy DNA (https://saasweb.hku.hk/EasyDNA/) was used to compute allelic frequencies, Observed Heterozygosity (Ho), Expected Heterozygosity (He), Power of Discrimination (PD), Probability of Exclusion (PE), and the Chi-square test statistic for Hardy Weinberg equilibrium. FORSTAT (https://fdl-uwc.shinyapps.io/forstat/) was used to calculate Match Probability and Polymorphism Information Content (PIC).

## Results

The observed allele frequencies for the 21 STR loci in the Minangkabau ethnic group in Indonesia are shown in Table 1. We found 162 alleles with a frequency range 0.02 - 0.36. The most frequent allele was 9 in TH01 loci. The most frequent allele types for each locus were: CSF1PO :12, TPOX: 11, TH01: 9, D13S317: 9, D16S539: 12, D18S51:14, D21S11: 30, D8S1179: 13, D7S820: 11, D5S818: 12, D3S1358: 15, FGA 22, vWA: 18, D10S1248: 15, D12S381: 20, D19S433: 13, D1S1656: 15, D22S1045: 16, D2S1338: 24, D2S441: 14, SE33: 28.2.

**Table 1:** Allele frequency for 21 loci Short Tandem Repeats (STR) Minangkabau ethnicity group, Indonesia

Allele	CSF1PO	TPOX	TH01	D13S317	D16S539	D18S51	D21S11	D8S1179	D7S820	D5S818	D3S1358	FGA	vWA
**6**	-	-	0.18	-	-	-	-	-	-	-	-	-	-
**7**	-	-	0.18	0.02	-	-	-	-	-	-	-	-	-
**8**	-	0.4	0.1	0.1	0.02	-	-	-	0.16	-	-	-	--
**9**	0.02	0.2	0.36	0.18	0.08	-	-	-	0.04	-	-	-	-
**9.3**	-	-	0.1	-	-	-	-	-	-	-	-	-	-
**10**	0.2	-	0.08	0.2	0.22	-	-	0.16	0.16	0.24	-	-	-
**11**	0.24	0.32	-	0.3	0.28	0.04	-	0.02	0.42	0.32	-	-	-
**12**	0.32	0.08	-	0.14	0.24	0.12	-	0.04	0.22	0.34	0.02	-	-
**13**	0.22	-	-	0.04	0.16	0.1	-	0.32	-	0.1	-	-	-
**14**	-	-	-	0.02	-	0.26	-	0.24	-	-	0.18	-	0.14
**15**	-	-	-	-	-	0.08	-	0.18	-	-	0.26	-	-
**16**	-	-	-	-	-	0.28	-	0.04	-	-	0.16	-	0.2
**17**	-	-	-	-	-	0.08	-	-	-	-	0.28	-	0.24
**18**	-	-	-	-	-	-	-	-	-	-	0.1	-	0.32
**19**	-	-	-	-	-	0.02	-	-	-	-	-	0.12	0.08
**20**	-	-	-	-	-	-	-	-	-	-	-	0.04	0.2
**21**	-	-	-	-	-	-	-	-	-	-	-	0.04	-
**22**	-	-	-	-	-	-	-	-	-	-	-	0.24	-
**23**	-	-	-	-	-	-	-	-	-	-	--	0.18	-
**24**	-	-	-	-	-	0.02	-	-	-	-	-	0.14	-
**24.2**	-	-	-	-	-	-	-	-	-	-	-	0.02	-
**24.3**	-	-	-	-	-	-	-	-	-	-	-	0.02	-
**25**	-	-	-	-	-	-	-	-	-	-	-	0.02	-
**26**	-	-	-	-	-	-	-	-	-	-	-	0.02	-
**27**	-	-	-	-	-	-	0.02	-	-	-	-	0.14	-
**28**	-	-	-	-	-	-	0.02	-	-	-	-	0.02	-
**29**	-	-	-	-	-	-	0.22	-	-	-	-	-	-
**30**	-	-	-	-	-	-	0.18	-	-	-	-	-	-
**30.2**	-	-	-	-	-	-	0.1	-	-	-	-	-	-
**31**	-	-	-	-	-	-	0.14	-	-	-	-	-	-
**31.2**	-	-	-	-	-	-	0.06	-	-	-	-	-	-
**32**	-	-	-	-	-	-	0.08	-	-	-	-	-	-
**32.2**	-	-	-	-	-	-	0.16	-	-	-	-	-	-
**33.2**	-	-	-	-	-	-	0.02	-	-	-	-	-	-

**Table:** 

Allele	D10S1248	D12S381	D19S433	D1S1656	D22S1045	D2S1338	D2S441	SE33
**8**	-	-	-	0.04	-	-	-	-
**10**	-	-	-	-	-	-	0.22	-
**11**	-	-	-	0.1	0.22	-	0.12	-
**11.2**	-	-	0.02	-	-	-	-	-
**11.3**	-	-	-	-	-	-	0.12	-
**12**	0.2	-	0.02	0.08	-	-	0.16	-
**12.3**	-	-	-	-	-	-	0.02	-
**13**	0.4	-	0.32	0.1	-	-	0.02	0.02
**13.2**	-	-	0.1	-	-	-	-	-
**14**	0.24	-	0.18	0.1	0.06	-	0.28	-
**14.2**	-	-	0.08	-	-	-	-	-
**15**	0.22	-	0.06	0.16	0.32	-	0.04	-
**15.2**	-	-	0.22	-	-	-	-	-
**15.3**	-	-	-	0.02	-	-	-	-
**16**	0.08	-	-	0.18	0.28	0.04	0.02	-
**17**	0.04	0.1	-	0.1	0.12	0.12	-	0.02
**17.3**	-	-	-	0.08	-	-	-	-
**18**	-	0.2	-	-	-	0.6	-	0.12
**18.3**	-	-	-	0.04	-	-	-	-
**19**	-	0.2	-	-	-	0.2	-	0.06
**20**	-	0.14	-	-	-	0.06	-	-
**21**	-	0.08	-	-	-	0.02	-	0.04
**22**	-	0.14	-	-	-	0.14	-	0.02
**22.2**	-	-	-	-	-	-	-	0.04
**23**	-	0.1	-	-	-	0.16	0.02	-
**24**	-	-	-	-	-	0.18	0.02	-
**24.2**	-	-	-	-	-	-	0.22	0.08
**25**	-	0.04	-	-	-	-	0.18	-
**25.2**	-	-	-	-	-	-	0.1	0.1
**26**	-	-	-	-	-	0.02	0.14	-
**26.2**	-	-	-	-	-	-	0.06	0.14
**27.2**	-	-	-	-	-	-	0.08	0.04
**28.2**	-	-	-	-	-	-	0.16	0.18
**29.2**	-	-	-	-	-	-	0.02	0.04
**30.2**	-	-	-	-	-	-	-	0.04
**31.2**	-	-	-	-	-	-	-	0.02
**32.2**	-	-	-	-	-	-	-	0.02
**33.2**	-	-	-	-	-	-	-	0.02

The variety of alleles is shown in table 2. The most common allele variety is at the SE33 loci, there are 17 types of alleles (13-33.2). The expected heterozygosity (He), power of discrimination, match probability, probability of exclusion, polymorphic information content, and chi-square for Hardy Weinberg equilibrium (HWE) for each locus were calculated in Table 3. The expected heterozygosity ranged from 0.691 (TPOX) to 0.904 (SE33). The power of discrimination ranges from 0.847 for the TPOX locus to 0.983 for the SE33 locus. The probability of exclusion ranges from 0.42 for the TPOX locus to 0.81 for the SE33 locus. Match probability ranges from 0.03 for the TPOX, D5S818, D22S1045 locus to 0.107 for the D2S441 locus. The polymorphic information content ranges from 0,610 (TH01) to 0.690 (D13S317). The combined power of discrimination in the Minangkabau population (CPD) was 92.43 % and the combined match probability (CMP) was 1.089 x 10^-28^. The chi-square test showed that all locus STR loci followed the Hardy Weinberg equilibrium (p > 0,05). All the loci are highly polymorphic (PIC >0,5). The TH01 locus is the fewest polymorphic STR loci (PIC = 0.610), and D13S317 and D19S433 are the most polymorphic (PIC=0.690).

**Table 3:** Variety of alleles 21 Short Tandem Repeats (STR) loci Minangkabau ethnicity group, Indonesia

Loci	Allele	Number of alleles
CSF1PO	9 - 13	5
TPOX	8 - 12	4
TH01	6 - 10	6
D13S317	7 - 14	8
D16S539	8 - 13	6
D18S51	11 - 23	9
D21S11	27 - 33.2	10
D8S1179	10 - 16	7
D7S820	8 - 12	5
D5S818	10 - 13	4
D3S1358	12 - 18	6
FGA	19 - 27	12
vWA	14 - 20	6
D10S1248	12 - 17	6
D12S381	17 - 25	8
D19S433	11.2 – 15.2	8
D1S1656	8 – 18.3	11
D22S1045	11 - 17	5
D2S1338	16 - 24	9
D2S441	10 - 16	9
SE33	13 – 33.2	17

**Table 3:** Expected Heterozygosity (He), Power of Discrimination (PD), Probability of exclusion (PE), Match Probability (MP), and Polymorphic Information Content (PIC) for 21 loci Short Tandem Repeats (STR) Minangkabau ethnicity group, Indonesia

Loci	He	PD	PE	MP	PIC
**CSF1PO**	0.751	0.894	0.52	0.004	0.673
**TPOX**	0.691	0.847	0.42	0.003	0.653
**TH01**	0.779	0.922	0.578	0.007	0.610
**D13S317**	0.806	0.936	0.622	0.008	0.690
**D16S539**	0.783	0.918	0.579	0.004	0.686
**D18S51**	0.814	0.942	0.64	0.015	0.681
**D21S11**	0.853	0.961	0.71	0.013	0.689
**D8S1179**	0.778	0.917	0.572	0.007	0.679
**D7S820**	0.722	0.881	0.48	0.006	0.642
**D5S818**	0.714	0.864	0.456	0.003	0.653
**D3S1358**	0.786	0.921	0.584	0.005	0.686
**FGA**	0.851	0.961	0.708	0.029	0.654
**vWA**	0.774	0.913	0.563	0.007	0.679
**D10S1248**	0.726	0.881	0.483	0.004	0.686
**D12S381**	0.853	0.961	0.71	0.009	0.665
**D19S433**	0.796	0.931	0.606	0.007	0.690
**D1S1656**	0.886	0.976	0.773	0.019	0.664
**D22S1045**	0.753	0.897	0.525	0.003	0.673
**D2S1338**	0.858	0.964	0.721	0.008	0.698
**D2S441**	0.816	0.942	0.642	0.107	0.658
**SE33**	0.904	0.983	0.81	0.047	0.661

## Discussion

The highest allele frequency was found in allele 9 at the TH01 locus (0.36). The high-frequency value indicates that the allele is common in the Minangkabau population. High frequency causes a decrease in the value of heterozygosity and power of discrimination. The TH01 locus is also the fewest polymorphic STR loci of the 21 loci used in this study.

The highest number of alleles was found at the SE33 loci. The highest expected heterozygosity, power of discrimination, and probability of exclusion were also found at the SE33 loci. The variety of alleles and their frequency influence the heterozygosity and power of discrimination. The heterozygosity and high power discrimination indicate that the locus is suitable for forensic identification.

One of the most polymorphic markers utilized in forensic human identification is the HUMACTBP2 (SE33) locus. There has never been any data on allele frequency at the SE33 loci in Indonesia. The findings of this study could serve as preliminary information on allele frequencies at the SE33 STR locus.

An analysis of the genetic variability of the SE33 locus by A. Barbaro concluded that the SE33 locus is effectively highly polymorphic and valuable not only for forensic identification but also in paternity cases (Barbaro *et al.*, 2015). In a study on the Kazakh population of Xinjiang in northwest China, SE33 loci showed the most significant power of discrimination. (Zhang *et al.*, 2016). A study by Jianye Ge, Arthur Eisenberg, and Bruce Budowle showed that the SE33 locus has the highest Average Kinship Index (AKI) compared to other loci (PentaE, D12S391, D1S1656, D2S1338, D18S51, FGA, D21S11, D8S1179, D7S820, vWA, D19S433, D13S317, D2S441, D3S1358, D10S1248, D16S539, TH01, CSF1PO, D5S818, DYS391, PentaD, D22S1045, and TPOX) (Ge, Eisenberg and Budowle, 2012).

The combined power of discrimination in the Minangkabau population (CPD) was 92.43 % and the combined match probability (CMP) was 1.089 x 10^-28^. All the loci are highly polymorphic (PIC >0,5). This indicates that these 21 loci can be used for forensic identification using STR in the Minangkabau population.

## Conclusion

The TH01 locus is an allele that is often found in the Minangkabau population. The SE33 locus had the highest heterozygosity, power discrimination and match probability among the 21 STR loci. All loci are polymorphic (p>0.5) so these 21 loci can be used for forensic identification and study of the Minangkabau population.

### Conflict of Interests

The authors declare that they have no competing interests.

**Abbreviations:**
CODIS -: Combined DNA Index System
STR -: Short Tandem Repeats
PD -: Power of Discrimination
MP -: Match Probability
PIC -: Polymorphic Information Content
Ho -: Observed Heterozygosity
He –: Expected Heterozygosity
PE -: Probability of Exclusion
